# Pollution Characteristics of Sb, As, Hg, Pb, Cd, and Zn in Soils from Different Zones of Xikuangshan Antimony Mine

**DOI:** 10.1155/2019/2754385

**Published:** 2019-09-08

**Authors:** Saijun Zhou, Andrew Hursthouse, Tengshu Chen

**Affiliations:** ^1^College of Civil Engineering, Hunan University of Science and Technology, Xiangtan, Hunan, China; ^2^Hunan Provincial Key Laboratory of Shale Gas Resource Utilization, Hunan University of Science and Technology, Xiangtan, Hunan, China; ^3^School of Computing, Engineering & Physical Sciences, University of the West of Scotland, Paisley PA1 2BE, UK; ^4^Department of Resources and Environmental Sciences, Quanzhou Normal University, Quanzhou, Fujian, China

## Abstract

Major sources of pollution during the antimony (Sb) mining and processing are mining waste rock, smelting waste, tailings dam, and underground tunnel wastewater. The aim of the present study was to assess magnitude of pollution from Sb mine by taking four types of samples: soil in the mining waste rock zone, soil in the smelting zone, soil in tailings zone, and soil in underground tunnel wastewater zone. Sixty soil samples from the four zones were taken for experimental work, and the contents and morphological characteristics of the six potentially toxic elements (PTEs) such as Sb, As, Hg, Pb, Cd, and Zn in the soil samples were measured by using a hydride generation atomic fluorescence spectrometer (AFS-9700). The results show that the soil of the mine area is seriously polluted. The average contents of Sb, As, Hg, Pb, Cd, and Zn in the soil reach 1267.20 mg·kg^−1^, 94.44 mg·kg^−1^, 1.46 mg·kg^−1^, 184.19 mg·kg^−1^, 8.54 mg·kg^−1^, and 1054.11 mg·kg^−1^, respectively. There exists good correlation between the PTEs in the soil, with Sb strongly positively correlated with As, Hg, Pb, and Zn. The intensity of pollution is highest in the antimony-smelting zone, where the potential ecological risk index is over 15,000, followed by the tailings zone and mining waste rock zone, with the underground tunnel wastewater zone being the lowest. Using sequential chemical extraction, the elements are associated with the residual fraction, followed by organic-sulfide fraction, and smaller portions in the Fe-Mn oxide, carbonate, and exchangeable fractions. There are great differences in the speciation content of different elements in different sampling zones. The study implicates that Sb-smelting zone is the potential source of PTEs and maximum metals are associated with residual phase, out of which significant portion is associated with mobile fraction or phase.

## 1. Introduction

Metal mining, mineral processing, and smelting have caused serious environmental pollution, which has attracted extensive attention from researchers [1–3]. The wastes produced during the process of mining, processing, and smelting not only occupy a large area, but also release a large number of harmful elements under the effect of rain leaching, resulting in continuous environmental pollution [4–7]. China is the world's largest producer of antimony, accounting for 80% of the world's total antimony output annually. Xikuangshan (XKS) Antimony Mine in Hunan Province is the only super-large antimony mine in the world. It has been mined and smelted for nearly 120 years, and its annual average output of antimony accounts for 25% of the global total [8]. Its longtime and large-scale mining and smelting have produced large amounts of antimony mine wastes. With surface runoff and precipitation infiltration, harmful elements like Sb and the associated elements As, Hg, and Zn have been released, threatening the environment and human beings [9–12]. When excessive amounts of antimony enter the body, they can cause diseases in the liver, skin, respiratory tract, and cardiovascular system and even lead to mutations and cancer [13, 14]. Therefore, antimony has been listed as a priority pollutant by the US Environmental Protection Agency and the European Union [15, 16]. Up to now, the researches are mainly focused on the leaching, migration, and transformation of metals in solid waste of mineral resources [17–19], and there are few studies on the characteristics of metal pollution in different functional mining zones. Previous studies have shown that the exploitation and smelting of antimony result in very serious Sb pollution in the soils of the surrounding mining areas [20], and antimony smelting slag is an important source of Sb pollution in nearby farmland soils [21]; the content of elements such as Sb, As, and Pb is usually very low in uncontaminated soils, but increases by three orders of magnitude in soils contaminated by antimony mining [11, 22], and the concentration of Sb in soils contaminated by abandoned mines ranges from 585 to 3184 mg·kg^−1^ [23]. Pollution occurs during the whole exploitation process (mining, mineral processing, and smelting), and each process presents different pollution characteristics; therefore, only a general study of potentially toxic element (PTE) pollution is not conducive to fully grasp the detail on the nature of information of heavy metal pollution. Therefore, taking XKS Sb mine as an example, this study makes a systematical investigation of the PTE pollution in the soil in different functional zones of the Sb mine, fully exposing its characteristics, aiming to provide theoretical support for soil pollution reduction and soil remediation.

## 2. Field Situation of Investigation

### 2.1. General Introduction

XKS Sb mine, known as the “World's Antimony Capital,” is located in Lengshuijiang City, Hunan Province, in central China (E111°18′57″∼E111°36′40″, N27°30′49″∼N27°50′38″). It borders Lianyuan City in the east, Xinshao County in the south, and Xinhua County in the west and north, with a mining area of 70 km^2^ and an Sb reserve of 400,000 tons. XKS Sb mine has two parts: the south mine (Wuhua, Feishuiyan) and the north mine (Laokuangshan, Tongjiayuan). The ores are simple in composition, with stibnite as the ore mineral. The gangue minerals include quartz and calcite, with minor amounts of fluorite, barite, and secondary gypsum. The alteration of the host limestone is dominated by silicification and subordinately by carbonatization and, to a lesser extent, by baritization and fluoritization [24]. Lengshuijiang has the continental monsoon climate in the subtropical zone, with the annual average temperature of 16.7°C, annual average rainfall of 1354 mm, and annual average relative humidity of 53.1%. The average temperature is 4.9°C in January and 28.2°C in July.

A large amount of waste rock, tailings, and smelting slags are produced during the Sb mining, processing, and smelting. These are partly used for filling the underground voids and piled up in the waste slack yard. The tailings produced in mineral processing are piped to the tailings dam located in the southwest of the mining area, which is located in the natural depression between two mountain bodies in the southwest of the mining area. Downstream is a large area of farmland, at risk from contamination from tailings release. The slags produced in the smelting process are partly placed in the waste slag yard, partly mixed, and piled with waste rocks in the open air. The south zone and the north zone of XKS Sb mine are exploited using a vertical shaft combined with different middle-level roadways. The drainage capacity of underground tunnel wastewater is 650 m^3^/d. The wastewater is pumped to the surface pond and discharged to the Lianxi River flowing through the mining area after simple treatment.

### 2.2. Division of Study Zone

Nonferrous metal mining activities mainly include mining, processing, and smelting, and during each process, solid, liquid, and gaseous wastes containing PTEs are produced. During the mining process, when minerals are broken, some metals are discharged to the earth surface through ventilation system along with the dust generated by drilling and blasting in underground mine; then they are deposited into soil and water through atmospheric diffusion, and some of them enter groundwater or surface water through tunnel wastewater. During the process of transportation of minerals underground or on the ground, the heavy metals in minerals enter the surrounding water and soil with scattering and dust. During mineral processing, a large number of tailings are produced and stored in the tailings dam. After simple treatment, the wastewater from mineral processing and tailings precipitation is recycled or used for irrigation of surrounding farmland, and some wastewater is discharged to the surrounding water body through the tailings dam drainage hole. At the same time, a large number of chemical agents (lead nitrate, xanthate, and terpineol) are used in the mineral processing of antimony, and most of them are metal complexing agents, which can be associated with ore deposit associated PTEs such as Sb, As, Hg, Pb, Cd, and Zn to make a complex pollution source from the site. The pollution caused by the exploitation of antimony mainly comes from the following sources: (1) mining waste rock, (2) smelting waste, (3) tailings dam, and (4) underground tunnel wastewater.

**Figure 1:**
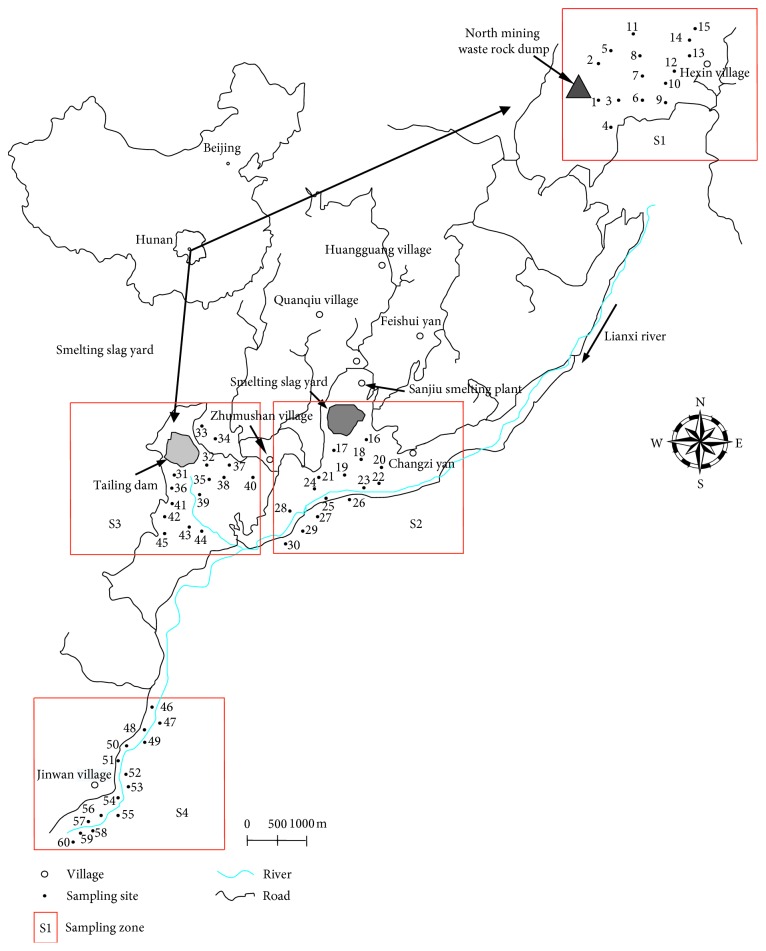
Simplified map of the study zone and sampling location of soil.

This study divides the XKS antimony mining area into four zones associated with the production process: mining waste stock zone (S1), smelting zone (S2), tailings zone (S3), and underground tunnel wastewater zone (S4). Following the topography of XKS Sb mine and the distribution of antimony ore waste (waste rock, smelting slags, and tailings), soil sampling was conducted in accordance with the requirements of the *Technical Specification for Soil Environmental Monitoring* (HJ/T 166-2004) [25], and a series of 15 soil samples were taken from each of the four soil contaminated areas. The sampling zones are shown in Figure 1.

## 3. Research Methods

### 3.1. Sample Collection and Processing

The four sampling zones (S1 to S4) are as follows: S1 is located in Hexin Village, about 2.5 km from the north mine; S2 is located in Changziyan Village near the south mine, less than 1 km from the smelter; S3 is located in Zhumushan Village near tail sand dam, about 1 km from tailings dam; and S4 is located in Jinwan Village in the lower reaches of Lianxi River, about 5.5 km from the tailings dam. The samples were collected by composite sampling where 100 g of soil was obtained from the plough layer (0–30 cm depth) of a 1 m^2^ from the center and at its four equidistant corners. A total of 15 soil samples were collected from each zone, giving a total of 60 samples. The soil samples collected in S1, S2, S3, and S4 are numbered 1–15, 16–30, 31–45, and 45–60, respectively. After being well mixed, the samples were put into the Teflon plastic sampling bags and taken back to the laboratory. Then, they were air-dried, cleaned of debris and gravel, ground with agate mortar, and sieved through a 100-mesh nylon sieve, and then kept in Teflon bags for later study.

### 3.2. Test and Analysis Methods

The soil samples were then treated as follows: 1.0 g of each soil sample was accurately weighed and placed in a 250 ml Erlenmeyer flask, heated on a hot plate (95 ± 5°C) for 10–15 minutes with 10 ml of 1 : 1 nitric acid added, and then cooled for 5 minutes and heated again for 5 minutes after 5 ml of concentrated nitric acid added. If brown fumes were observed, the sample had been oxidized by nitric acid. This procedure was repeated (add 5 ml of concentrated nitric acid each time), until brown fumes were absent. The soil sample was then cooled, with the addition of 2 ml of ultrapure water (Shijiazhuang Tester TST-UPWC-30) and 3 ml of 30% H_2_O_2_, and then heated again. When no reaction in the flask was observed, the sample was added with more 30% H_2_O_2_, 1 ml at each time, and heated until the appearance of the samples did not change, and heated again on the hot plate (95 ± 5°C) for 2 hours and cooled. Finally, ultrapure water was added to the samples to make a volume of 100 ml and then got filtered. The content of the six elements in the soil was measured with hydride generation atomic fluorescence spectrometer AFS-9700 (Beijing Haitian). The pH value of soil was determined by glass electrode method, with the soil-to-water ratio of 2.5 : 1.

The chemical association of PTEs in soil determines their toxicity to organisms, and the proportion of each metal in the fractions is the main influencing factor [26]. The sequential extraction method based on the method of Tessier et al. [27, 28] was applied to the samples and measured by a hydride generation atomic fluorescence spectrometer. The chemical fraction and reagents that were used in the sequential extraction procedure are briefly summarized as follows: exchanged fraction (F1): “exchangeable,” 1 M MgCl_2_ at pH 7 for 1 h; carbonate fraction (F2): “bound to carbonate mineral,” 1 M sodium acetate adjusted to pH with acetic acid for 5 h at room temperature; reducible fraction (F3): “bound to amorphous Fe and Mn hydroxides,” 0.04 M hydroxylamine hydrochloric acid in 25% (*v*/*v*) acetic acid at 96°C for 6 h; organic and sulfide fraction (F4): “bound to organic matter and sulfide minerals,” 30% hydrogen peroxide and 0.02 M nitric acid at 85°C for 5 h; and residual fraction (F5): “residual,” 5 : 1 mixture of nitric acid and perchloric acid, followed by evaporation to dryness and dissolution of the residue in 6N nitric acid. F1 is the most mobile fraction and can become readily available to plants and to the other living organisms. F2 is sensitive to pH change, and lowering of pH helps in the release of metal cation from this fraction. F3 supposedly signifies that each metal bound to iron and manganese oxides might be released if the soil is subjected to reductive environment. F4 reflects the quantity of metal bound to the organic matter and organic sulfurs, which can become mobile in oxidative condition. F5 is safe for the environment as it is released under very extreme condition like pH < 2 [29]. Different fractions of heavy metals in the soil will affect the availability of heavy metals to plants. Therefore, heavy metals can be classified into three forms: easy-to-use state, moderate-to-use state, and inert state. Exchanged fraction (F1) can be easily absorbed by plants. Carbonate fraction (F2), reducible fraction (F3), and organic and sulfide fraction (F4) can be utilized by plants, while residual fraction (F5) cannot be absorbed and utilized by plants [30].

### 3.3. Quality Control and Statistical Analyses

In order to ensure the accuracy of the data and the stability of the testing instrument, the soil standard reference material (GBW07406) from the National Institute of Metrology (China) was digested and measured in the same way as the soil collected in the field. The test recovery rates of Sb, As, Hg, Pb, Cd, and Zn were 95%–106%, 94%–107%, 94–104%, 97%–105%, 95%–104%, and 96%–105%, respectively. At the same time, in each batch of analytical samples, reagent blanks were added, and 10% of the samples were determined repeatedly. The relative standard deviation (RSD) of repeated determination was less than 10%.

SPSS 17.0 was used for the statistical analysis of the average value of PTEs in soil and their correlation, and Origin 9.0 was adopted to draw the figures.

## 4. Results and Discussion

### 4.1. Distribution of PTEs in Soils in Different Sampling Zones

Based on the analysis and determination of the collected soils, the range and mean values of soil pH and PTEs content in different zones are shown in Table 1.

It can be seen from Table 1 that there are significant differences in the content of PTEs in four different soil sampling zones. The difference is mainly reflected in antimony and zinc, especially in the soil of antimony smelting waste yard, where the highest contents of Sb and Zn are 3947.68 mg·kg^−1^ and 3297.79 mg·kg^−1^, respectively. There is no significant difference in the contents of As, Hg, Pb, and Cd in the soils of each sampling zone. The method of potential ecological hazard index (RI_*j*_) is used to evaluate the pollution degree of PTEs in each sampling zone [31, 32], for it can define the relationship between the ecological hazard coefficient *E*_*j*_^*i*^ and the potential ecological hazard index (RI) and the degree of ecological hazard [33]. The calculation of RI_*j*_ is as follows:

(1) $$\begin{array}{c} E_{j}^{i}=T_{i}\times C_{j}^{i}=T_{i}\times \frac{C_{\text{m}}}{C_{\text{r}}},\\ {\text{RI}}_{j}=\overset{n}{\underset{i=1}{\sum }}E_{j}^{i}, \end{array}$$

where *E*_*j*_^*i*^ is the index of a single potential ecological risk of PTE *i* at sampling point *j*; RI_*j*_ is the index of comprehensive potential ecological risk at sampling point *j*; *T*_*i*_ is the toxic response coefficient of PTE *i* (Sb = 40, As = 30, Hg = 10, Pb = 10, Cd = 5, and Zn = 1) [34], which reflects the level of toxicity and the sensitivity of organisms to the pollution; *C*_*j*_^*i*^ is the pollution coefficient of PTE *i* at sampling point *j*; *C*_m_ is the measured value of PTE *i* at sampling point *j*; and *C*_r_ is the reference content of PTE *i*, and *n* represents the total kinds of PTEs (*n* = 6).

The reference value used affects the classification of samples using comprehensive potential ecological risk index. He et al. [35] took PTEs contents of polluted zones background samples as reference. Liao and Wu [36] and Cheng and Zhou [37] used the highest background value before modern industrialization as reference. This paper takes the soil background value of Hunan Province as reference.

Comprehensive potential ecological risk index is listed in Table 1. It can be seen that the four sampling zones are seriously polluted by PTEs. The order of pollution intensity in different zones from heavy to light is smelting pollution zone, tailings pollution zone, mine waste rock pollution zone, and mine drainage zone.

**Figure 2:**
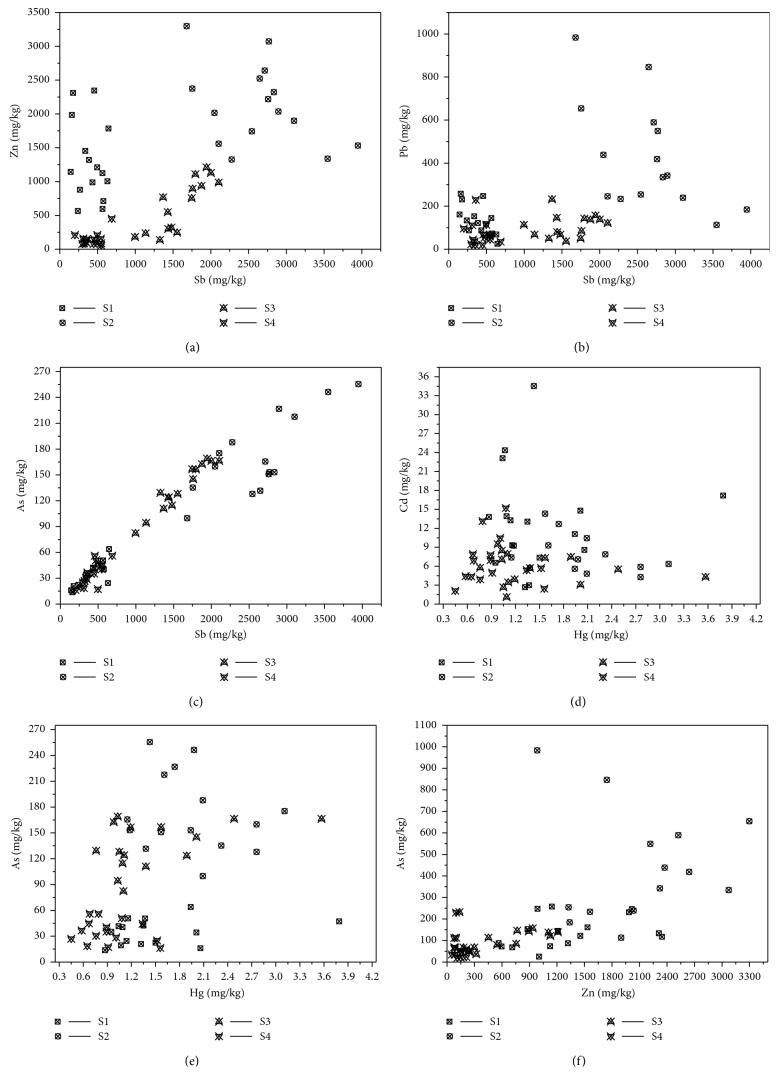
Correlation of PTEs contents in soil samples of different polluted zones.

Figure 2 shows the results of comparison in all samples. It is easy to see that each zone is characterized by Sb-Zn pollution as its main product is Sb-Zn. The crude ores of XKS Sb mine are mainly stibnite and sphalerite. The grade of antimony and zinc is 4.25%, and the grade of Sb and zinc concentrate is 53.20% and 45.57% [38], respectively. The pollution caused by Sb and Zn is the most serious in each zone, especially in antimony-smelting zone. The waste residue, wastewater, and waste gas from antimony smelting process released a large amount of PTEs and polluted the surrounding soil.

The correlation coefficient between PTEs contents and pH value in antimony mine soil is shown in Table 2. It shows that pH is negatively correlated with Sb and As; Sb is positively correlated with As, Hg, Pb, and Zn; As is positively correlated with Hg, Pb, and Zn; Hg is positively correlated with Pb; Hg is positively correlated with Zn; Pb is positively correlated with Zn; Cd is not significantly correlated with pH and other PTEs. The soil around XKS Sb mine is polluted by Sb, As, Hg, Pb, and Zn, which indicates that the soil around XKS Sb mine is polluted by PTEs in the compound form. This is also in line with the characteristics of soil pollution in nonferrous metal mining areas in China, that is, the pollution of PTEs is mostly comprehensive or compound.

### 4.2. Specific Distribution of PTEs in Soils in Different Sampling Zones

**Figure 3:**
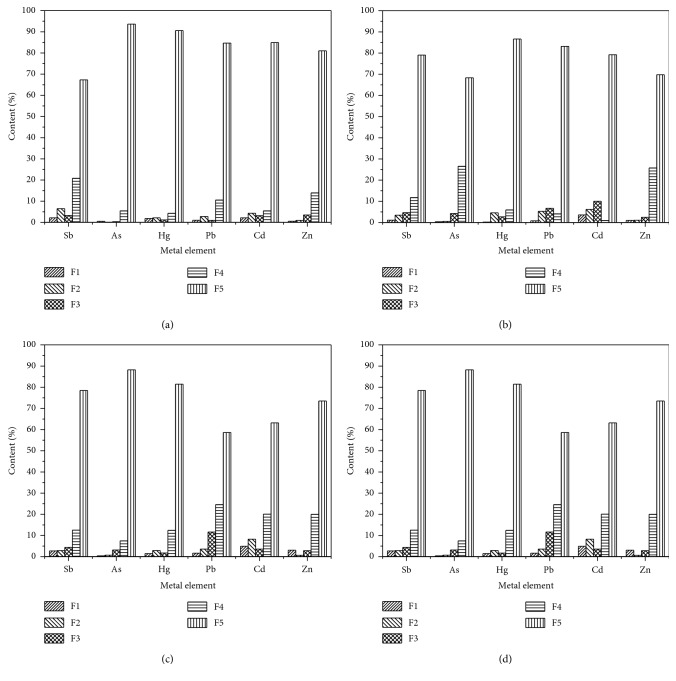
Content of Sb, As, Hg, Pb, Cd, and Zn in different polluted zones (*n* = 60): (a) S1; (b) S2; (c) S3; (d) S4; F1: exchanged fraction; F2: carbonate fraction; F3: reducible fraction; F4: organic and sulfide fraction; F5: residual fraction.

A comparison of the percentages of Sb, As, Hg, Pb, Cd, and Zn in different sampling zones was made and is shown in Figure 3. It can be seen that the residual fraction is the main form of PTEs in the soil of each sampling zone, followed by the organic and sulfide fraction, and the other three fractions of PTEs occupy the least proportion. The proportion of PTEs in each fraction in the four sampling zones was in the order of residual fraction > organic-sulfide fraction > Fe-Mn oxide fraction > carbonate fraction > exchangeable fraction, and there were significant differences in the content of various forms of PTEs in different sampling zones, with the exchangeable fraction the most significant [11, 12, 39, 40]. The ratio of the exchangeable fraction of Sb and Cd in the four sampling zones is relatively high, and that of Zn, Pb, and As in soil is relatively low, but the absolute content of these three elements is relatively high, so they have great potential hazards. In addition, the carbonate fraction, Fe-Mn oxide fraction, and organic-sulfide fraction are sensitive to environmental conditions and can supply more mobile fraction of the PTEs contents when these are modified (acid rain, microorganisms, and weathering) [41, 42]. The sum of the exchangeable fraction, carbonate fraction, Fe-Mn oxide fraction, and organic-sulfide fraction for PTEs in the soil around the antimony ore slag stacking zone accounts for 22.3% of the total. This therefore requires sensitive monitoring and control.

## 5. Conclusions

The soil in the sampling zones of XKS Sb mine shows strong enrichment in a number of PTEs associated with the mining activity. The average content of Sb, As, Hg, Pb, Cd, and Zn in the soils of XKS Sb mine is 1267.20 mg·kg^−1^, 94.44 mg·kg^−1^, 1.46 mg·kg^−1^, 184.19 mg·kg^−1^, 8.54 mg·kg^−1^, and 1054.11 mg·kg^−1^, respectively, which are, respectively, 425.23, 6.75, 16.22, 6.14, 108.10, and 11.10 times of the background values of the soils of Hunan Province. There are strong correlations between the PTEs: Sb is especially significantly positively correlated with As, Hg, Pb, and Zn, indicating that the five elements are associated with primary extraction processes. According to potential ecological hazard index RI_*j*_, the four sample zones vary in risk with the order of smelting zone > tailings zone > mining waste rock zone > tunnel wastewater zone. Their RI_*j*_ index is 15235.45, 9494.99, 7024.28, and 4779.33, respectively, and highlights the need for the Sb-smelting zone to be prioritized in management of pollution, for it is an important place for PTEs release and migration to the environment. While the highest portion of PTEs content is associated with residual fraction, a significant portion is associated with mobile fraction or phases sensitive to changing environmental conditions.

## Figures and Tables

**Table 1:** Comparison of PTEs contents in soil sample of different polluted zones.

Polluted zone	Number of samples	pH	Content of PTEs (mg·kg^−1^)						RI_*j*_
			Sb	As	Hg	Pb	Cd	Zn	
S1	15	6.47∼7.34	143.74∼645.33	16.03∼63.85	0.87∼3.79	24.86∼257.88	2.68∼24.36	564.28∼2345.09	7024.28
S2	15	4.78∼7.26	1679.54∼3947.68	99.85∼255.51	1.15∼3.11	112.97∼983.28	4.81∼34.54	1326.31∼3297.79	15235.45
S3	15	5.04∼7.08	995.67∼2103.65	56.22∼168.86	0.76∼3.57	36.47∼231.65	1.09∼9.47	179.60∼1209.48	9494.99
S4	15	5.77∼7.98	199.66∼687.04	16.45∼56.22	0.45∼1.56	19.54∼229.95	2.11∼15.21	54.76∼452.31	4779.33
*M*		6.44	1267.20	94.44	1.46	184.19	8.54	1054.11	
*S*			2.98	14	0.09	27	0.079	95	
*M*/*S*			425.23	6.75	16.22	6.14	108.10	11.10	

*M:* average value; *S:* soil background value in Hunan Province.

**Table 2:** Correlation matrix of PTEs in sampling zones (*n* = 60).

PTEs	pH	Sb	As	Hg	Pb	Cd	Zn
pH	1						
Sb	−0.505^*∗∗*^	1					
As	−0.544^*∗∗*^	0.955^*∗∗*^	1				
Hg	−0.131	0.362^*∗∗*^	0.384^*∗∗*^	1			
Pb	−0.125	0.471^*∗∗*^	0.338^*∗∗*^	0.279^*∗*^	1		
Cd	0.045	0.084	0.053	0.010	−0.024	1	
Zn	−0.015	0.536^*∗∗*^	0.423^*∗∗*^	0.415^*∗∗*^	0.811^*∗∗*^	0.202	1

Significance level: ^*∗∗*^*P* < 0.01 and ^*∗*^*P* < 0.05.

## Data Availability

The data in this paper are from field sampling and laboratory testing, and the experimental results are shown in the attached table. We can ensure that the data are authentic and credible and that the data can be adopted by other readers.
